# Supplementary material for: Substrate-specific effects of natural genetic variation on proteasome activity
Source: PLoS Genet. 2023 May 1;19(5):e1010734. doi: 10.1371/journal.pgen.1010734 (PMC10174532; doi:10.1371/journal.pgen.1010734)
Supplement: Attachment — Submitted filename: proteasome_QTL_reviewer_response.pdf [file pgen.1010734.s006.pdf]

Responses to Reviewers

Reviewer 1

Collins et al. have taken advantage of the high genetic diversity between two distinct Saccharomyces cerevisiae yeast strains, BY and RM, and used it with a pair of tandem fluorescent timer (TFT) ubiquitin-independent degron fusions to identify QTLs that affect proteasome-mediated degradation rates of the two TFT reporters. They identify 15 QTLs, 12 of which have substrate-specific effects. They use this information together with data from a highly similar study (also deposited in bioRxiv by the same group) to identify potential genes responsible for the QTL effects. One possible hit is in the intergenic region upstream of RPT6, which encodes a regulatory subunit of the proteasome. They identify a single nucleotide polymorphism and using CRISPR/Cas9 to change this residue in a BY background to the RM base pair and find that this is sufficient to moderately increase degradation of a number of the reporters (N-end rule and one of the ubiquitin-independent reporters).

Reviewer 1 Major Comments

This is an interesting study as far as it goes, and the experimental analysis is solid overall. I confess to being a little disappointed that it did not fully follow through on at least the apparent upstream RPT6 hit.

We thank the reviewer for their thoughtful evaluation of our work and constructive feedback. The revised manuscript contains multiple follow-up experiments to further characterize the causal nucleotide at RPT6 *-175. We establish that the variant increases* RPT6 *expression without affecting* ALG13 *expression and that* RPT6 *overexpression is sufficient to increase the degradation of multiple substrates with which we detect the QTL containing* RPT6 *-175.*

One could also argue that this manuscript and the aforementioned bioRxiv paper should be combined, especially since data from the latter paper are used extensively here, but this is not my call.

We respectfully disagree and argue that the current paper adds new and important insights into how genetic variation shapes proteasome activity. Three key findings from our now-published previous study were: (1) genetic influences on the UPS are widespread, (2) loci affecting the UPS rule typically do so in substrate-specific manner, and (3) that this substrate specificity results, in

part, from multiple large-effect causal variants in ubiquitin system genes that target distinct N-end Rule substrates[1].

These results raised three important questions that were not addressed in our previous study: (1) are genetic effects on the proteasome as equally numerous as those on the UPS, (2) are genetic influences on proteasome activity similarly substrate-specific, and (3) what are the mechanisms by which causal variants shape proteasome activity. The current manuscript addresses all three of these critical questions, contains new experimental results, and provides, to our knowledge, the first genome-wide view of how natural genetic variation influences proteasome activity.

I found the high fraction of substrate-specific QTLs a little surprising. Among the 15 QTLs, I would expect multiple proteasome subunit or assembly factor hits, and it is hard to see how the proffered explanation for the 12 substrate-specific ones –that recognition or binding of these two substrates involves different proteasome subunits– can fully account for such specificity. The fact that MKT1 and IRA2 were likely hits makes one think that many/most of the QTLs will turn out to affect proteasomal degradation in highly indirect ways, which while in all likelihood are real effects, will be difficult to understand more deeply.

We did not intend to suggest that effects on individual proteasome subunits are the sole explanation for the high degree of substrate-specificity in our proteasome activity QTLs. Accordingly, we have substantially revised the Discussion section (page 27, paragraphs 2 and 3, line 465) to emphasize that effects on individual proteasome subunits are one of potentially many mechanisms that could explain the substrate specificity of proteasome activity QTLs and provide additional examples of other mechanisms that could create substrate-specific effects on proteasome activity.

To put the reviewer's comment regarding proteasome genes in context, we cataloged sequence differences between the BY and RM strains in all genes, proteasome genes, and E3 ubiquitin ligase genes, using the latter as a protein turnover-relevant gene category for comparison to proteasome genes. Reviewer Response Figure 1 shows that proteasome genes are depleted of total BY / RM variants when compared to E3 ligase genes (Wilcoxon $p = 0.015$*) and of missense variants when compared to all genes in the yeast genome (Wilcoxon* $p = 6.5e\text{-}5$*) or when compared to E3 ligases (Wilcoxon* $p = 1.1e\text{-}7$*). Thus, there is a limited reservoir of proteasome gene variants with the potential to directly alter proteasome function.*

Given this relative lack of variation in proteasome genes, we agree with the reviewer's interpreta-

tion that much of the genetic basis of variation in proteasome activity will involve indirect effects. This notion is consistent with recent theoretical[2] *and empirical*[3,4] *observations suggesting that most trait heritability is driven by weak* trans-*acting variation in non-core (i.e., non-proteasome for proteasome activity) genes. Indeed, we argue that this is a novel and important result in the manuscript: We show that most heritable variation in proteasome activity likely results from individual genetic differences in non-proteasome genes. Consequently, understanding how genetic effects on the proteasome contribute to variation in traits such as health, aging, and disease will require understanding how these indirect genetic effects influence the proteasome. We believe our manuscript is an important first step in this regard.*

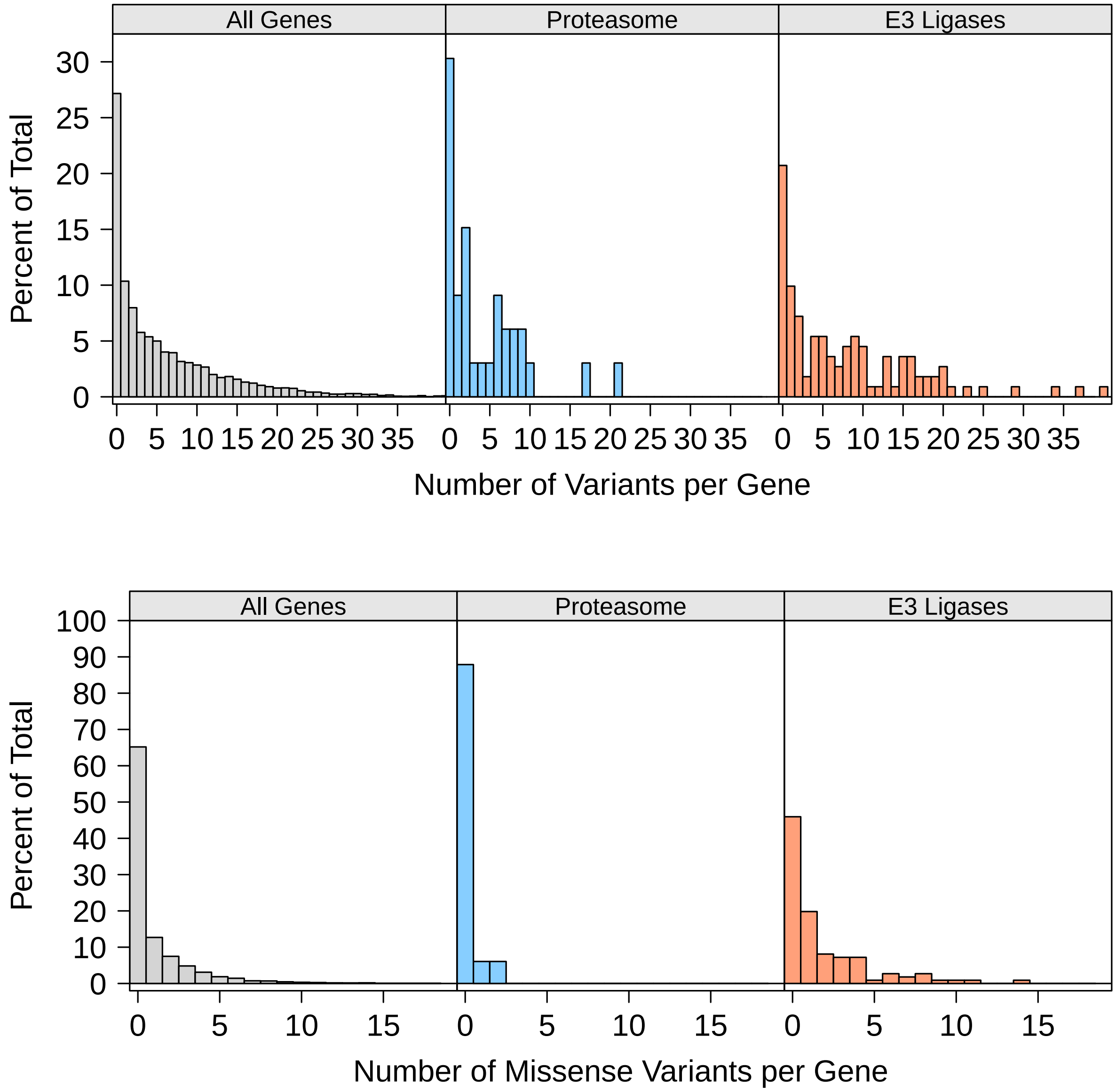

Reviewer Response Figure 1: *Histograms of the total number of BY / RM variants per gene (top)*

and BY / RM missense variants per gene (bottom) separated by category.

Reviewer 1 Minor Comments

Some additional comments:

Line 41 "Ubiquitin system enzymes bind degradation-promoting signal sequences" – generally, only the E3 enzymes do.

To better convey the functions of ubiquitin system enzymes, the phrase mentioned by the reviewer is replaced with the following in the revised manuscript (page 4, para. 1, line 43):

"Ubiquitin system enzymes target proteins for degradation by binding degradation-promoting signal sequences (termed "degrons") and covalently attaching chains of the small protein ubiquitin."

Line 57: "Until recently, it was largely unknown how individual genetic differences affect UPS protein degradation" There are lots of genetic data on genetic differences that affect the UPS. I think what is meant here is the question of how natural or semi-natural genetic variation affects the UPS. This should be clarified.

The reviewer's interpretation is correct and the revised manuscript (page 4, para. 2, line 59) includes the following sentence that more clearly articulates the indicated sentence's meaning:

"Until recently, it was largely unknown how natural genetic variation affects UPS protein degradation."

Line 88: "proteasome can exist in multiple configurations" What exactly is meant by this phrase? Please define 'configuration'.

The revised manuscript (page 5, para. 3, line 92) includes the following paragraph that clarifies the meaning of the phrase mentioned by the reviewer by providing an expanded review of the literature:

"Proteasomes can also be assembled in multiple configurations that impart distinct affinities for different classes of substrates. In yeast, the catalytically active 20S core particle may be uncapped or singly or doubly capped with the 19S regulatory particle or the proteasome activator Blm10[5]*. 20S proteasomes capped with 19S regulatory particles have high affinity for polyubiquitinated proteins*[6–8]*. In contrast, uncapped 20S proteasomes preferentially degrade unfolded or intrinsically disordered substrates that have not been ubiquitinated*[9–11]*. Blm10-capped 20S proteasomes prefer-*

entially degrade short peptides, rather than proteins[12–14]. Genetic effects on the composition of the "proteasome pool"[15] could, therefore, create substrate-specific changes in protein degradation."

Line 99: "when using these [ubiquitin reporter] systems to map genetic influences on UPS activity, variant effects on the ubiquitin system may mask or obscure specific effects on proteasomal protein degradation." Not clear why this should be the case. For example, if a UPS substrate is used as a reporter, why would variants in proteasome subunits not be detected? I would expect to see both ubiquitin conjugation enzyme and proteasome allelic variations unless the latter specifically have a lower relative impact. Please clarify.

The revised manuscript (page 6, para. 1, line 106) expands on the sentence mentioned by the reviewer to more clearly articulate how variant effects on the ubiquitin system could obscure variant effects on the proteasome:

"However, the output of these reporters reflects the activities of both the ubiquitin system and the proteasome, potentially hindering detection of variants that specifically affect the proteasome. In particular, QTLs often span dozens of genes and report the composite signal of multiple linked variants. As a result, the effect of a causal variant may not be reflected in a QTL if its effect direction is opposite one or more additional causal variants with larger effects. Multiple lines of evidence suggest that QTL regions often contain multiple causal variants[1,16–18]. In our previous work, four QTL regions we fine-mapped to causal ubiquitin system genes each contained multiple causal variants and were, along with QTLs containing HAP1 *and* MKT1*, the most frequently detected and largest effect size QTLs for the N-end Rule[1]. This suggests that variant effects on the ubiquitin system could mask or obscure specific effects on the proteasome when mapping with UPS activity reporters. Therefore, approaches that can directly and specifically measure proteasome activity are needed to understand the genetics of this trait."*

Line 216: "Ac/N-degrons are generated and recognized by a common set of molecular effectors. Reflecting this, many QTLs for Ac/N-degrons affect all or a majority of the full set of Ac/N-degrons. By contrast, Arg/N-degrons are created and recognized via molecular mechanisms that affect individual or small subsets of Arg/N-degrons." I don't follow this reasoning: each class requires a common set of factors (E1, E2s, E3s) and has a small number of processing enzymes specific to certain degrons: for Ac/N-degrons, some will require MAPs (not sure how the N-end substrates were made for this study though) and different ones will require different Ac transferases, while for Ac/N-degrons, a very small number will require a deamidase and a small number

will require Arg transferase.

We have substantially revised the text mentioned by the reviewer to better explain why we expected the sets of QTLs affecting Arg/N-degrons to be more substrate-specific than those affecting Ac/N-degrons (page 16, para. 2, line 237):

"The N-end Rule is divided into two primary branches based on how N-degrons are generated and recognized[19–22]. Based on the molecular mechanisms of Arg/N-degron processing and recognition, we hypothesized that QTLs affecting Arg/N-degrons would have predominantly substrate-specific effects. For example, 2 of 12 Arg/N-degrons require deamidation by Nta1[22] and 4 of 12 Arg/N-degrons require arginylation by Ate1[23]. QTLs affecting Nta1 or Ate1 would, therefore, be expected to influence, at most, 17% and 33% of Arg/N-degrons. Similarly, the E3 ligase Ubr1 has multiple binding sites and QTLs affecting these sites or their allosteric regulation[24] would also be expected to affect only a subset of Arg/N-degrons[1]. In contrast, Doa10, the E3 ligase for Ac/N-degrons, has a single RING-CH-type finger domain that has not been shown to differentially target distinct Ac/N-degrons. Similarly, the NatA complex acetylates 4 of 8 Ac/N-degrons[25], suggesting a potentially higher rate of QTL sharing for Ac/N-degrons compared to Arg/N-degrons. Consistent with these predictions, we previously showed that variation at NTA1 *and* UBR1 *differentially affects Arg/N-degrons, while variation at* DOA10 *and the Ac/N-degron E2 ubiquitin-conjugating enzyme* UBC6 *affects Ac/N-degrons similarly[1]."*

Line 264: "multiple factors specifically regulate the degradation of ubiquitin-independent proteasomal substrates, without affecting the degradation of ubiquitinated substrates (ref. 80)." Please give an example or two.

In the sentence mentioned by the reviewer, we were specifically referring to factors, such as Elp2 or Ncs6, that have been shown to regulate the ubiquitin-independent turnover of proteasomal substrates but do not affect the turnover of ubiquitinated substrates[26,27]. However, we do not know if these or similar genes are causal for our proteasome activity QTLs and have therefore removed the indicated sentence from the revised manuscript.

Line 318: "The Rpn4 degron, in particular, is recognized by distinct 19S regulatory particle receptors from the other substrates tested here (Ref. 64) and may, therefore, be unaffected by RPT6 -175." Ref. 64 only tested the Rpn4 degron; similar mapping has not been done for ODC degron

binding to my knowledge. It could be that the ODC degron is bound to the same subunits.

The sentence mentioned by the reviewer is removed from the revised manuscript.

Line 421: "Ref 97 showed overexpression of alpha1, not alpha3/Pre9, has these effects. There is also work in human cells showing alpha4/PSMA7 overexpression can enhance proteasome activity and lead to enhanced heavy metal resistance (Padmanabhan et al. 2016).

We thank the reviewer for alerting us to this error, which we have corrected in the revised manuscript. We have also added additional examples that show that increasing proteasome subunit expression increases proteasome activity, including Padmanabhan et al. 2016 (page 29, para. 2, line 523).

Line 427: "Thus, PAAF1 association with Rpt6 creates a stable Rpt6 pool that can be used to rapidly drive proteasome assembly." This doesn't really address the question of how increasing levels of one subunit of the ˜30+ subunit proteasome complex drives assembly of the whole particle. There are some examples of how this can work, but none involve Rpt6 to my knowledge.

The revised manuscript (page 29, para. 3, line 539) contains an expanded discussion of how increasing RPT6 *expression could increase proteasome activity:*

While increasing proteasome subunit expression is thus an established means of increasing proteasome activity, the mechanism(s) of this effect are not well-understood. They may involve coordinated increases in the expression of additional proteasome genes or enhanced proteasome assembly. In the case of RPT6*, one possibility is that increasing expression levels increases the number of 19S regulatory particles and, in turn, the fraction of 26S proteasomes. The proteasome pool comprises uncapped 20S core particle and 20S singly or doubly capped with 19S regulatory particles ("26S proteasomes") or other proteasome activators such as Blm10. Estimates of the fraction of uncapped 20S proteasomes in the proteasome pool vary across species and cell types, but are generally no less than 30%[11, 15, 28, 29], suggesting that a large fraction of the proteasome pool could be converted to 26S proteasomes. Moreover, the 26S fraction is dynamic and responsive to changes in 19S subunit expression. For example, in human cells, decreasing the expression of either of the 19S subunits Rpt6 and Rpn2 reduces the fraction of 26S proteasomes[30]. Current models of proteasome assembly posit that the 20S core particle can serve as a template for assembling the 19S regulatory particle[31–33]. Rpt6 plays a critical role in this process - insertion of its C-terminal tail into the $\alpha2$-$\alpha3$ pocket is the first step in assembling the 19S regulatory particle's base that sits atop the 20S core particle[32–34]. After insertion, Rpt6 functions as an anchor to which other*

RPT heterodimers are added[32–34]*. These findings suggest that increasing* RPT6 *expression could increase the 26S proteasome fraction by promoting the formation of an assembly intermediate that acts as a scaffold for further 19S assembly onto the 20S core.*

Experimentally, it should be straightforward to examine the RPT6 hit more carefully using the CRISPR'ed allele created. First, does the SNP really affect only RPT6 transcription and not the nearby ALG13 gene, to which it is actually closer? The latter gene is involved in an essential biological process, protein glycosylation, and indirect effects from changes of its expression could certainly impact the protein homeostasis network (for example, endoplasmic reticulum-associated protein degradation).

In the revised manuscript, we present data showing that the causal RPT6 *-175 variant increases Rpt6 abundance but does not affect Alg13 (page 20, para., 4, line 348 and Figure 6C / D.)*

Second, the authors make the interesting speculation that because the RM strain is from a "European wine" lineage, enhanced proteasome activity might be selected for its possibly enhanced ethanol tolerance. This could be tested experimentally with the CRISPR'd and matched BY strain too.

We have removed the text suggesting that RPT6 *-175 may have arisen as a result of adaptation to the wine-making environment from the revised manuscript. We retain figure 8A to provide readers with a view of how the causal* RPT6 *-175 RM allele alters the molecular properties of the* RPT6 *promoter.*

Third, the SNP is proposed to allow Yap1 binding in the RM but not BY strain. The dependence of increased RPT6 mRNA on Yap1 in the CRISPR'd strain can be measured.

We have removed text from the revised manuscript suggesting that the increase in Rpt6 results from Yap1 binding to the RPT6 *promoter.*

Finally, if the SNP increases Rpt6 levels, the prediction of the authors is that there will be increased proteasomes as a result. This is also testable (although it might be beyond the expertise of a genetics-intensive lab, so I would not demand this).

We did not perform this experiment because it is outside of the laboratory's expertise. We thank the reviewer for not requiring this experiment as part of the revision.

In summary, it would be important, in my opinion, to show that at least one QTL (RPT6-175)

directly affects proteasome activity or regulation in a way that can be understood at least roughly in mechanistic terms.

In the revised manuscript we demonstrate that the RPT6 *-175 variant increases Rpt6 levels without affecting Alg13 levels. We further show that overexpressing* RPT6 *expression is sufficient to increase the degradation of multiple UPS substrates that we used to detect the QTL containing* RPT6 *-175. Collectively, these results provide strong support for our interpretation that RPT6 -175 increases proteasome activity by increasing Rpt6 levels.*

Reviewer 2

In this manuscript Collins and coworkers examine the influence of natural genetic variation in yeast on the stability of two degrons (as a proxy for proteasome activity). To do so they leverage fluorescence protein timers, where steady-state ratiometric measurements of fluorescence are related to the stability of each reporter. Using flow cytometry they perform bulk segregant analysis and map over a dozen loci that are linked to differences in reporter stability. Many such linkages appear to selectively influence one reporter but not the other. Finally, using CRISPR-Cas9 approaches, the authors demonstrate that one of the alleles they identified can recapitulate the effects they identified statistically. Overall this is a fine manuscript and the data seem sound. Additional experiments, analyses, and qualifications are needed (described below) to support or clarify specific claims. Likewise, there are multiple opportunities to improve impact and generalizability.

We thank the reviewer for their positive feedback and thoughtful suggestions to improve the manuscript. As described below, we have used these comments to conduct additional analyses and incorporate additional text that will increase the impact and generalizability of the revised manuscript.

Reviewer 2 Major comments

1. Controls for the assay. A couple of points are important to discuss or test explicity. First, as it is present in **the** key transcriptional regulator of the proteosome, does the present of additional Rpn4 degron copies influence the UPS?

The Rpn4 TFT contains the N-terminal 80 amino acids of the yeast Rpn4 protein. This domain functions as a ubiquitin-independent degron, but does not contain transcriptionally active DNA binding domains[35–37]*. We, therefore, interpret the reviewer's comment as suggesting that addi-*

tional Rpn4 degron molecules resulting from the Rpn4 TFT expression could compete with endogeneous Rpn4 for proteasome 19S subunit receptor binding and, in so doing, increase proteasome activity through endogeneous Rpn4 stabilization. To address this question, we created BY strains harboring the Rpn4 TFT driven by the ACT1 *or* TDH3 *promoters. Previous work has shown that GFP levels can be used to estimate the TFT's abundance in cells*[38]*. Using this approach, we observed that the* TDH3 *promoter produced a nearly-4 fold increase in the Rpn4 TFT's abundance as compared to the Rpn4 TFT expressed from the* ACT1 *promoter (Reviewer Response Figure 2A). However, we found no difference in proteasome activity between BY strains harboring the TFT driven by the* ACT1 *versus* TDH3 *promoters (Reviewer Response Figure 2B), suggesting that extra copies of the Rpn4 degron do not affect proteasome activity. We acknowledge that we cannot rule out that expressing the Rpn4 TFT via the* ACT1 *promoter alters UPS activity relative to strains without any TFT and that no further alteration then occurs in response to the greatly increased expression conferred by the TDH3 promoter, but do not think that this is likely.*

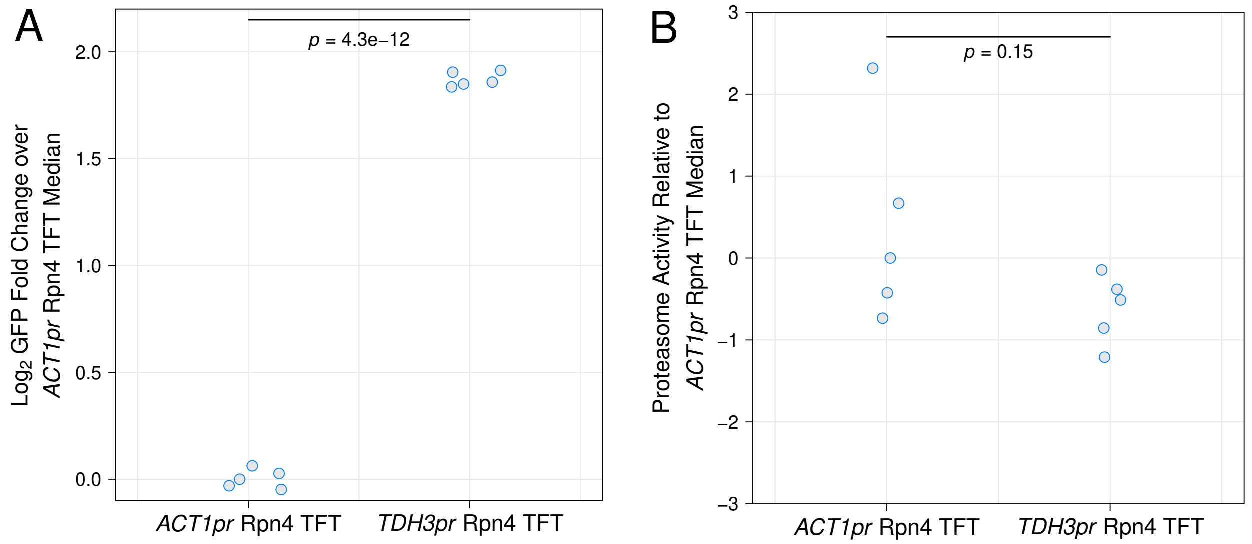

Reviewer Response Figure 2: *Proteasome activity at different levels of Rpn4 TFT expression. A. sfGFP levels were used to estimate the difference in TFT expression between BY strains expressing the Rpn4 TFT from the* ACT1 *or* TDH3 *promoter. B. The TFT ratio was used to measure proteasome activity in the same strains. In A. / B., all values are Z-score normalized to the median of the BY strain expressing the Rpn4 TFT from the* ACT1 *promoter*

Likewise, controls for growth phase, cell shape, size, adhesion, and other properties that might influence turnover are important. It may not be possible to fully account for all confounding

variables, but they should at least be commented upon.

Our protocols for fluorescence-activated cell sorting and flow cytometry gate cells on the basis of forward and side scatter to restrict cell populations to haploid cells of the same approximate size, shape, and, by extension, cell cycle phase. This gating approach also eliminates any aggregates of adherent cells from analysis. This information in included in the Methods section (page 36, para. 2, line 715).

2. Comparison to prior literature for these degrons. The FP timers enable single cell resolution and impressive scale. At the same time, they report on steady-state fluorescent ratios rather than actual kinetic proteasome function. How do the differences between the OTC and RPN4 degrons measured here compare to conventional measurements of their proteasomal turnover?

Previous studies determined that the half-life of the mouse ODC degron in yeast is approximately 6 minutes[39]*. The half-life of the N-terminal ubiquitin-independent Rpn4 degron has not, to our knowledge, been precisely determined, but appears to be between 10 and 20 minutes*[40,41]*. We observed that the steady state ODC TFT ratio was approximately half that of the Rpn4 TFT , suggesting each reporter's output provides an accurate estimate of its degradation kinetics. Because we are unable to obtain a precise estimate of the half-life of the Rpn4 ubiquitin-independent degron, we have not included this information in the revised manuscript. We*[1] *and others*[27,42] *have also shown that the TFT ratio accurately reports a protein's degradation kinetics by comparing the TFT ratio to the well-characterized half-lives of the 20 possible N-degrons of the UPS N-end rule*[1,27]*. Previous work also demonstrated that the TFT ratio provides greater sensitivity and dynamic range compared to conventional measures of protein degradation by either cycloheximide chase or pulse-chase labeling*[27]*.*

Likewise, do the ratios obtained with this assay behave as one might expect when titrating proteasome inhibitors etc.?

We have used gene deletions to characterize how the TFT ratio changes in response to perturbations that alter ubiquitin proteasome system activity here and elsewhere[1]*. We observe that the TFT ratio reports the expected stabilization of both N-degrons and ubiquitin-independent degrons in response to deleting* RPN4 *and the expected branch-specific stabilization of N-degrons in response to deleting* UBR1 *or* DOA10*. Others have shown that the TFT ratio increases as expected in*

response to bortezomib treatment[43].

Finally, it is critical that the manuscript include some discussion of the quantitative ranges of difference in degradation rates that can be observed, and an estimation of those that cannot, based on the maturation kinetics of the GFP and RFP variants used.

The revised Results section contains information on the quantitative ranges of difference in degradation rates that can be observed with the superfolder GFP / mCherry TFT:

"Our TFTs contained the faster-maturing green fluorescent protein (GFP[44]*) superfolder GFP (sfGFP) and the slower-maturing red fluorescent protein (RFP*[45]*) mCherry (Figure 2A). The two fluorophores in the TFT mature at different rates and, as a result, the RFP / GFP ratio changes over time. If the TFT's degradation rate is faster than the RFP's maturation rate, the TFT's output, expressed as the* $-\log_2$ *RFP / GFP ratio, is directly proportional to its degradation rate (Figure 2B). The superfolder GFP / mCherry tandem fluorescent timer can measure the degradation of substrates with half-lives ranging from several minutes to several hours*[27]*, making it an ideal reporter system for studying short-lived proteasomal substrates."*

The revised Discussion section elaborates on which proteins can be studied using the superfolder GFP / mCherry TFT:

"Based on these results, we anticipate that variant effects on the degradation of individual proteins will also be highly substrate-specific. Understanding how natural genetic variation affects the proteome through effects on the degradation of individual proteins will thus require reporters that can sensitively measure the degradation of proteins with half-lives ranging from several minutes to several hours[46,47]*. The mCherry / sfGFP TFT is well-suited to this purpose. Previous studies have shown that this TFT should be suitable for measuring the degradation of approximately 80% of yeast proteins based on their half-lives*[27,27]*, assuming the protein tolerates the TFT tag. Recently, a genome-wide TFT tagging approach successfully used the mCherry / sfGFP timer to measure the turnover of approximately 70% (around 4,000 proteins) of the yeast proteome*[48]*, suggesting that degradation QTLs for most proteins could be mapped using this reporter. TFTs with red fluorescent proteins that mature over longer time scales, such as mRuby or dsRed, can be used to measure the degradation of longer-lived proteins*[27]*."*

3. Comparison to known genetic architecture of the UPS and other core regulatory processes. Others have examined UPS function with deletion and overexpression experiments, among other

approaches. How do the results here compare in terms of number of genes identified, overlap, etc.?

It is difficult to directly compare our results with those obtained using genome-wide deletion or overexpression-based approaches. A critical difference between screening approaches and the genetic mapping method we employed is that genetic mapping detects the effects of natural genetic variation. As we show in Reviewer Response Figure 1, there is a limited source of natural variation in proteasome genes that could directly affect proteasome activity. Instead, we find many QTL regions contain genes with no known links to the proteasome. We believe this is an important insight from our work, since it suggests that genetic effects on the proteasome that contribute to variation in traits such as health, longevity, and disease will arise through small, indirect effects, as we elaborate on the in the Discussion section (page 26, para. 2, line 433).

Do the authors find essential genes that were not previously seen?

Answering this question comprehensively would require experimental dissection of causal genes, as we have performed for the (known) essential proteasome component Rpt6. We note that several QTLs did not contain any genes with known roles in proteasome function or assembly, suggesting unknown components or, perhaps more likely, indirect effects, as described in the revised Results section (page 19, para. 2, line 299).

Did prior studies also find substrate specific genetic contributions?

Two previous proteome-wide efforts found multiple substrate-specific changes in the proteome following deletion or perturbation of UPS genes[38,49]*. These works appear as references 13 and 14 in the revised manuscript.*

And how do the number and effect sizes of QTLs here compare to those seen for other traits examined by MPRA by both the PI and others in the field?

We detected 11 QTLs with the ODC TFT and 7 QTLs with the Rpn4 TFT. These values are quite similar to the number of loci found for other genetically complex traits in this cross of yeast strains. Previous studies using the BY / RM cross detected a mean of 6.5 eQTLs per gene[50]*, 7.5 pQTLs per gene*[51]*, 7.5 QTLs per N-degron*[1]*, and a mean of 11 QTLs across 17 diverse chemical resistance traits*[52]*. Using the absolute allele frequency difference between high and low pools as a measure of effect size, we observe that N-degron and proteasome activity QTLs have similar effects (N-degron QTL mean = 0.19, proteasome activity QTL mean = 0.15, Mann-Whitney U* $p = 0.097$*).*

Collectively, these results suggest that genetic effects on the proteasome and ubiquitin system are similarly numerous.

4. Completeness of the genetic dissection and origins of degron specific differences. How much of the difference between BY and RM can be explained by the loci that the authors identified? Also, how much of the specificity in mapping for each degron is a simple property of their different stabilities to begin with? From the investigator's prior work with N-end rule reporters and GFP fusions it might be possible (and interesting) to speculate on how, quantitatively, this type of regulation may integrate with other types of trans regulatory variation.

We cannot readily calcuate the amount of variance explained by our proteasome activity QTLs as explained in the revised Discussion (page 28, para. 3, line 506). It is also difficult to precisely determine how reporter stability influences QTL substrate specificity. Less stable reporters allow greater selection strength between the high and low proteasome activity pools, which may influence QTL detection power[53]. However, as discussed in the revised manuscript (page 17, para. 2, line 261), we observe multiple substrate-specific QTLs for more stable reporters here (e.g., VIIc and XV) and in our prior study[1], suggesting that QTL substrate-specificity does not simply reflect differences in reporter dynamic range.

5. Tie up the mechanism. I commend the allele reconstruction experiment, but it also left me wondering about the proposed mechanism, which is presently based almost entirely on speculation. First, although altered regulation of Rpt6 is obviously the easier explanation, the authors should do an experiment to exclude Alg13. Second, seeing allele-specific rescue with Yap1 overexpression or increased Rpt6 ovexpression would signficantly strengthen their case. Of course there can be challenges with such experiments, but they are simple and well worth trying.

In the revised manuscript, we present results showing that the causal RPT6 *-175 variant increases Rpt, but not Alg13 levels. We further show that overexpression of* RPT6 *increases the degradation of multiple substrates with which we detect the QTL containing RPT6 -175 (page 20, para. 4, line 353). These results provide strong support for our conclusion that* RPT6 *-175 increases proteasome activity by increasing* RPT6 *expression.*

6. Ecological impact. The authors determine that the RM allele is derived, which seems reasonable. But at the same time in the tree in Fig. S1, which I would recommend moving to the main text, there are several niches that also have a high frequency of the allele. It may challenging to

distinguish positive from balancing selection, but it does look like there may be some enrichment for fermentative niches? I would recommend that the authors investigate this possibility.

To investigate a potential enrichment of the RM allele of RPT6 *-175 in fermentative niches, we computed the allele's frequency across the clades defined in Peter et al., 2018*[54] *and classified clades as "fermentative" or "non-fermentative". Reviewer Response Figure 3 shows the results of this analysis. We did not detect enrichment of the RM allele of* RPT6 *-175 in fermentative niches versus non-fermentative niches (Mann-Whitney U* p *= 0.25). This is likely due, in part, to the low overall number of clades with the RM* RPT6 *-175 allele (16 of 37, 43%). Consistent with this observation, the population frequency of the RM* RPT6 *-175 allele drops from 37.7% to 18.3% if the "Wine / European" clade is not considered. Thus, our results suggest that the enrichment of RM* RPT6 *-175 in the "Wine / European" clade is largely specific to this group of strains.*

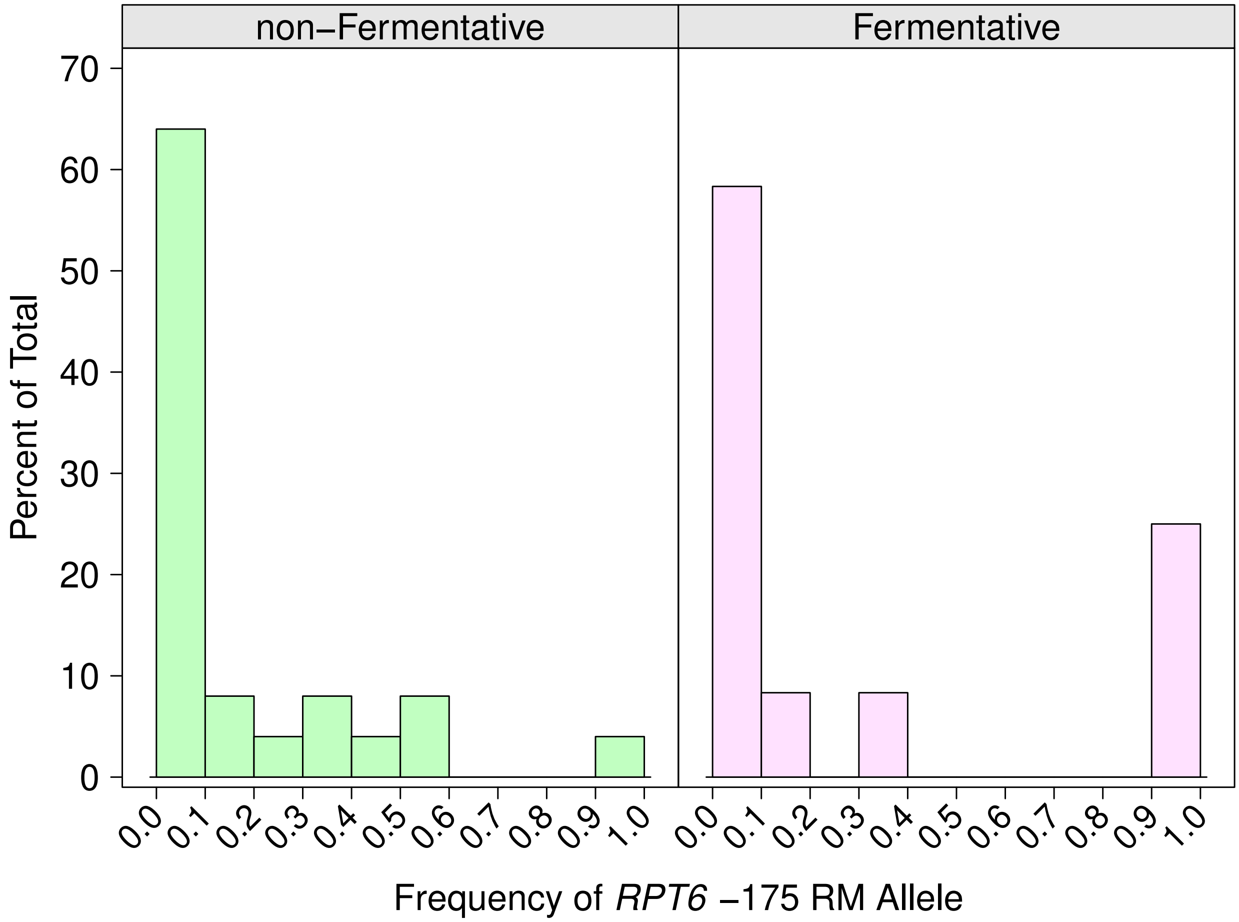

Reviewer Response Figure 3: *Histograms of the frequency of the RM* RPT6 *-175 allele in non-fermentative versus fermentative clades.*

Reviewer 2 Minor Comments

7. In my view, Figure 1 can probably go to the supplement, whereas Figure S1 should probably be in the main text.

We appreciate the reviewer's suggestion and acknowledge that Figure 1 is not needed in the main text for readers in the field of proteasomal protein degradation. However, we anticipate that this work will also be of interest to readers in the field of complex trait genetics, in particular, individuals studying the genetics of gene expression. Based on discussions in our laboratory, we are confident that these readers will appreciate the visual distinction between ubiquitin-dependent and -independent proteasomal protein degradation Figure 1 provides and have, therefore, left this figure in the main text in the revised manuscript.

Per the reviewer's suggestion, Figure S1 appears as part of main text Figure 8 in the revised manuscript.

8. For all quantitative statements I would ask the authors to provide not only the p-value and statisical test used but also the magnitude of the effect.

The revised manuscript includes effect sizes for all statistical comparisons.

9. What happened with the outlier in Fig. 2E?

We cannot explain the BY rpn4Δ *ODC TFT outlier Figure 2E. This strain may have acquired a compensatory mutation in response to the deleterious effect of deleting the* RPN4 *gene. However, we do not have data supporting this notion and we therefore continue to include data from this strain in Figure 2E in the revised manuscript.*

10. Limitations and advantages of the BSA approach should be discussed more fully. For example epistasis might be very hard to see, etc.

The revised Discussion section (page 28, para. 3, line 507) describes the advantages and limitations of bulk segregant analysis:

"We identified QTLs for proteasome activity using bulk segregant analysis, an approach that has previously been used to characterize the genetic basis of variation in a host of molecular, cellular, and organismal traits[52, 55–58]*. By assaying large numbers of individuals, bulk segregant analysis provides high statistical power to detect variant effects on a trait*[52, 57]*. Here, we used high-*

throughput reporters to measure proteasome activity in millions of recombinant progeny from a cross of the BY and RM strains, which allowed us to reproducibly identify proteasome activity QTLs. Moreover, bulk segregant analysis is efficient in terms of time, labor, and resources as compared to linkage or association mapping. In particular, by generating two "bulks" with extreme phenotypes, we could detect proteasome activity QTLs through pooled whole-genome sequencing, rather than genotyping individual meiotic progeny. However, the choice of bulk segregant analysis also involves limitations that arise from this pooled whole-genome sequencing approach. Because we do not ascertain the genotypes of individual meiotic progeny, we cannot readily estimate the heritability of proteasome activity or the variance explained by the QTLs we detect. For the same reason, we are unable to detect genetic interactions between loci. Recent advances[59] could enable efficient, statistically powerful mapping of proteasome activity using individual meiotic progeny in future studies, which would address these limitations."

11. Can the authors please add columns for the number of genes and polymorphisms within each confidence interval described in Table 1?

The revised manuscript includes Supplementary Table 5, which contains the number of genes and polymorphisms in each confidence interval in Table 1.

References

[1] M. A. Collins, G. Mekonnen, and F. W. Albert. Variation in ubiquitin system genes creates substrate-specific effects on proteasomal protein degradation. *Elife*, 11, Oct 2022.

[2] E. A. Boyle, Y. I. Li, and J. K. Pritchard. An Expanded View of Complex Traits: From Polygenic to Omnigenic. *Cell*, 169(7):1177–1186, Jun 2017.

[3] X. Liu, Y. I. Li, and J. K. Pritchard. Trans Effects on Gene Expression Can Drive Omnigenic Inheritance. *Cell*, 177(4):1022–1034, May 2019.

[4] W. Zhang, G. R. Reeves, and D. Tautz. Testing Implications of the Omnigenic Model for the Genetic Analysis of Loci Identified through Genome-wide Association. *Curr Biol*, 31(5):1092–1098, Mar 2021.

[5] E. Kish-Trier and C. P. Hill. Structural biology of the proteasome. *Annu Rev Biophys*, 42:29–49, 2013.

[6] V. Chau, J. W. Tobias, A. Bachmair, D. Marriott, D. J. Ecker, D. K. Gonda, and A. Varshavsky. A multiubiquitin chain is confined to specific lysine in a targeted short-lived protein. *Science*, 243(4898):1576–1583, Mar 1989.

[7] D. Finley. Recognition and processing of ubiquitin-protein conjugates by the proteasome. *Annu Rev Biochem*, 78:477–513, 2009.

[8] J. S. Thrower, L. Hoffman, M. Rechsteiner, and C. M. Pickart. Recognition of the polyubiquitin proteolytic signal. *EMBO J*, 19(1):94–102, Jan 2000.

[9] J. M. Baugh, E. G. Viktorova, and E. V. Pilipenko. Proteasomes can degrade a significant proportion of cellular proteins independent of ubiquitination. *J Mol Biol*, 386(3):814–827, Feb 2009.

[10] L. S. Hsieh, W. M. Su, G. S. Han, and G. M. Carman. Phosphorylation regulates the ubiquitin-independent degradation of yeast Pah1 phosphatidate phosphatase by the 20S proteasome. *J Biol Chem*, 290(18):11467–11478, May 2015.

[11] F. Kumar Deshmukh, D. Yaffe, M. A. Olshina, G. Ben-Nissan, and M. Sharon. The Contribution of the 20S Proteasome to Proteostasis. *Biomolecules*, 9(5), May 2019.

[12] T. Dange, D. Smith, T. Noy, P. C. Rommel, L. Jurzitza, R. J. Cordero, A. Legendre, D. Finley, A. L. Goldberg, and M. Schmidt. Blm10 protein promotes proteasomal substrate turnover by an active gating mechanism. *J Biol Chem*, 286(50):42830–42839, Dec 2011.

[13] V. Ustrell, L. Hoffman, G. Pratt, and M. Rechsteiner. PA200, a nuclear proteasome activator involved in DNA repair. *EMBO J*, 21(13):3516–3525, Jul 2002.

[14] B. M. Stadtmueller and C. P. Hill. Proteasome activators. *Mol Cell*, 41(1):8–19, Jan 2011.

[15] A. V. Morozov and V. L. Karpov. Proteasomes and Several Aspects of Their Heterogeneity Relevant to Cancer. *Front Oncol*, 9:761, 2019.

[16] N. S. Abell, M. K. DeGorter, M. J. Gloudemans, E. Greenwald, K. S. Smith, Z. He, and S. B. Montgomery. Multiple causal variants underlie genetic associations in humans. *Science*, 375(6586):1247–1254, 03 2022.

[17] S. Lutz, K. Van Dyke, M. A. Feraru, and F. W. Albert. Multiple epistatic DNA variants in a single gene affect gene expression in trans. *Genetics*, 220(1), 01 2022.

[18] K. Renganaath, R. Cheung, L. Day, S. Kosuri, L. Kruglyak, and F. W. Albert. Systematic identification of cis-regulatory variants that cause gene expression differences in a yeast cross. *Elife*, 9, Nov 2020.

[19] A. Varshavsky. The N-end rule pathway and regulation by proteolysis. *Protein Sci.*, 20(8):1298–1345, Aug 2011.

[20] C. S. Hwang, A. Shemorry, and A. Varshavsky. N-terminal acetylation of cellular proteins creates specific degradation signals. *Science*, 327(5968):973–977, Feb 2010.

[21] A. Bachmair, D. Finley, and A. Varshavsky. In vivo half-life of a protein is a function of its amino-terminal residue. *Science*, 234(4773):179–186, Oct 1986.

[22] R. T. Baker and A. Varshavsky. Yeast N-terminal amidase. A new enzyme and component of the N-end rule pathway. *J Biol Chem*, 270(20):12065–12074, May 1995.

[23] R. G. Hu, C. S. Brower, H. Wang, I. V. Davydov, J. Sheng, J. Zhou, Y. T. Kwon, and A. Varshavsky. Arginyltransferase, its specificity, putative substrates, bidirectional promoter, and splicing-derived isoforms. *J Biol Chem*, 281(43):32559–32573, Oct 2006.

[24] G. C. Turner, F. Du, and A. Varshavsky. Peptides accelerate their uptake by activating a ubiquitin-dependent proteolytic pathway. *Nature*, 405(6786):579–583, Jun 2000.

[25] M. Perrot, A. Massoni, and H. Boucherie. Sequence requirements for Nalpha-terminal acetylation of yeast proteins by NatA. *Yeast*, 25(7):513–527, Jul 2008.

[26] M. A. Hoyt, S. McDonough, S. A. Pimpl, H. Scheel, K. Hofmann, and P. Coffino. A genetic screen for Saccharomyces cerevisiae mutants affecting proteasome function, using a ubiquitin-independent substrate. *Yeast*, 25(3):199–217, Mar 2008.

[27] A. Khmelinskii, P. J. Keller, A. Bartosik, M. Meurer, J. D. Barry, B. R. Mardin, A. Kaufmann, S. Trautmann, M. Wachsmuth, G. Pereira, W. Huber, E. Schiebel, and M. Knop. Tandem fluorescent protein timers for in vivo analysis of protein dynamics. *Nat. Biotechnol.*, 30(7):708–714, Jun 2012.

[28] B. Fabre, T. Lambour, L. Garrigues, M. Ducoux-Petit, F. Amalric, B. Monsarrat, O. Burlet-Schiltz, and M. P. Bousquet-Dubouch. Label-free quantitative proteomics reveals the dynamics of proteasome complexes composition and stoichiometry in a wide range of human cell lines. *J Proteome Res*, 13(6):3027–3037, Jun 2014.

[29] I. Sahu, S. M. Mali, P. Sulkshane, C. Xu, A. Rozenberg, R. Morag, M. P. Sahoo, S. K. Singh, Z. Ding, Y. Wang, S. Day, Y. Cong, O. Kleifeld, A. Brik, and M. H. Glickman. The 20S as a stand-alone proteasome in cells can degrade the ubiquitin tag. *Nat Commun*, 12(1):6173, Oct 2021.

[30] P. Tsvetkov, M. L. Mendillo, J. Zhao, J. E. Carette, P. H. Merrill, D. Cikes, M. Varadarajan, F. R. van Diemen, J. M. Penninger, A. L. Goldberg, T. R. Brummelkamp, S. Santagata, and S. Lindquist. Compromising the 19S proteasome complex protects cells from reduced flux through the proteasome. *Elife*, 4, Sep 2015.

[31] R. J. Tomko and M. Hochstrasser. Molecular architecture and assembly of the eukaryotic proteasome. *Annu Rev Biochem*, 82:415–445, 2013.

[32] S. Park, J. Roelofs, W. Kim, J. Robert, M. Schmidt, S. P. Gygi, and D. Finley. Hexameric assembly of the proteasomal ATPases is templated through their C termini. *Nature*, 459(7248):866–870, Jun 2009.

[33] S. Park, X. Li, H. M. Kim, C. R. Singh, G. Tian, M. A. Hoyt, S. Lovell, K. P. Battaile, M. Zolkiewski, P. Coffino, J. Roelofs, Y. Cheng, and D. Finley. Reconfiguration of the proteasome during chaperone-mediated assembly. *Nature*, 497(7450):512–516, May 2013.

[34] V. Sokolova, F. Li, G. Polovin, and S. Park. Proteasome Activation is Mediated via a Functional Switch of the Rpt6 C-terminal Tail Following Chaperone-dependent Assembly. *Sci Rep*, 5:14909, Oct 2015.

[35] D. Ju, H. Xu, X. Wang, and Y. Xie. The transcription activation domain of Rpn4 is separate from its degrons. *Int J Biochem Cell Biol*, 42(2):282–286, Feb 2010.

[36] D. S. Karpov, V. V. Tutyaeva, and V. L. Karpov. Mapping of yeast Rpn4p transactivation domains. *FEBS Lett*, 582(23-24):3459–3464, Oct 2008.

[37] G. Mannhaupt, R. Schnall, V. Karpov, I. Vetter, and H. Feldmann. Rpn4p acts as a transcription factor by binding to PACE, a nonamer box found upstream of 26S proteasomal and other genes in yeast. *FEBS Lett*, 450(1-2):27–34, Apr 1999.

[38] K. E. Kong, B. Fischer, M. Meurer, I. Kats, Z. Li, F. Rühle, J. D. Barry, D. Kirrmaier, V. Chevyreva, B. J. San Luis, M. Costanzo, W. Huber, B. J. Andrews, C. Boone, M. Knop, and A. Khmelinskii. Timer-based proteomic profiling of the ubiquitin-proteasome system reveals a substrate receptor of the GID ubiquitin ligase. *Mol Cell*, 81(11):2460–2476, 06 2021.

[39] A. Henderson, J. Erales, M. A. Hoyt, and P. Coffino. Dependence of proteasome processing rate on substrate unfolding. *J Biol Chem*, 286(20):17495–17502, May 2011.

[40] S. W. Ha, D. Ju, and Y. Xie. The N-terminal domain of Rpn4 serves as a portable ubiquitin-independent degron and is recognized by specific 19S RP subunits. *Biochem Biophys Res Commun*, 419(2):226–231, Mar 2012.

[41] E. L. Reichard, G. G. Chirico, W. J. Dewey, N. D. Nassif, K. E. Bard, N. E. Millas, and D. A. Kraut. Substrate Ubiquitination Controls the Unfolding Ability of the Proteasome. *J Biol Chem*, 291(35):18547–18561, Aug 2016.

[42] I. Kats, A. Khmelinskii, M. Kschonsak, F. Huber, R. A. Knieß, A. Bartosik, and M. Knop. Mapping Degradation Signals and Pathways in a Eukaryotic N-terminome. *Mol Cell*, 70(3):488–501, 05 2018.

[43] H. Zhang, E. Linster, L. Gannon, W. Leemhuis, C. A. Rundle, F. L. Theodoulou, and M. Wirtz. Tandem Fluorescent Protein Timers for Noninvasive Relative Protein Lifetime Measurement in Plants. *Plant Physiol*, 180(2):718–731, Jun 2019.

[44] J. D. Pedelacq, S. Cabantous, T. Tran, T. C. Terwilliger, and G. S. Waldo. Engineering and characterization of a superfolder green fluorescent protein. *Nat. Biotechnol.*, 24(1):79–88, Jan 2006.

[45] N. C. Shaner, R. E. Campbell, P. A. Steinbach, B. N. Giepmans, A. E. Palmer, and R. Y. Tsien. Improved monomeric red, orange and yellow fluorescent proteins derived from Discosoma sp. red fluorescent protein. *Nat. Biotechnol.*, 22(12):1567–1572, Dec 2004.

[46] A. Belle, A. Tanay, L. Bitincka, R. Shamir, and E. K. O'Shea. Quantification of protein half-lives in the budding yeast proteome. *Proc Natl Acad Sci U S A*, 103(35):13004–13009, Aug 2006.

[47] R. Christiano, N. Nagaraj, F. Frohlich, and T. C. Walther. Global proteome turnover analyses of the Yeasts S. cerevisiae and S. pombe. *Cell Rep*, 9(5):1959–1965, Dec 2014.

[48] A. Khmelinskii, E. Blaszczak, M. Pantazopoulou, B. Fischer, D. J. Omnus, G. Le Dez, A. Brossard, A. Gunnarsson, J. D. Barry, M. Meurer, D. Kirrmaier, C. Boone, W. Huber, G. Rabut, P. O. Ljungdahl, and M. Knop. Protein quality control at the inner nuclear membrane. *Nature*, 516(7531):410–413, Dec 2014.

[49] R. Christiano, H. Arlt, S. Kabatnik, N. Mejhert, Z. W. Lai, R. V. Farese, and T. C. Walther. A Systematic Protein Turnover Map for Decoding Protein Degradation. *Cell Rep*, 33(6):108378, 11 2020.

[50] F. W. Albert, J. S. Bloom, J. Siegel, L. Day, and L. Kruglyak. Genetics of trans-regulatory variation in gene expression. *Elife*, 7, 07 2018.

[51] F. W. Albert, S. Treusch, A. H. Shockley, J. S. Bloom, and L. Kruglyak. Genetics of single-cell protein abundance variation in large yeast populations. *Nature*, 506(7489):494–497, Feb 2014.

[52] I. M. Ehrenreich, N. Torabi, Y. Jia, J. Kent, S. Martis, J. A. Shapiro, D. Gresham, A. A. Caudy, and L. Kruglyak. Dissection of genetically complex traits with extremely large pools of yeast segregants. *Nature*, 464(7291):1039–1042, Apr 2010.

[53] F. Duveau, B. P. Metzger, J. D. Gruber, K. Mack, N. Sood, T. E. Brooks, and P. J. Wittkopp. Mapping small effect mutations in Saccharomyces cerevisiae: impacts of experimental design and mutational properties. *G3 (Bethesda)*, 4(7):1205–1216, Apr 2014.

[54] J. Peter, M. De Chiara, A. Friedrich, J. X. Yue, D. Pflieger, A. Bergström, A. Sigwalt, B. Barre, K. Freel, A. Llored, C. Cruaud, K. Labadie, J. M. Aury, B. Istace, K. Lebrigand, P. Barbry, S. Engelen, A. Lemainque, P. Wincker, G. Liti, and J. Schacherer. Genome evolution across 1,011 Saccharomyces cerevisiae isolates. *Nature*, 556(7701):339–344, 04 2018.

[55] R. W. Michelmore, I. Paran, and R. V. Kesseli. Identification of markers linked to disease-resistance genes by bulked segregant analysis: a rapid method to detect markers in specific genomic regions by using segregating populations. *Proc Natl Acad Sci U S A*, 88(21):9828–9832, Nov 1991.

[56] F. W. Albert, D. Muzzey, J. S. Weissman, and L. Kruglyak. Genetic influences on translation in yeast. *PLoS Genet.*, 10(10):e1004692, Oct 2014.

[57] I. M. Ehrenreich, J. P. Gerke, and L. Kruglyak. Genetic dissection of complex traits in yeast: insights from studies of gene expression and other phenotypes in the BYxRM cross. *Cold Spring Harb Symp Quant Biol*, 74:145–153, 2009.

[58] C. Brion, S. M. Lutz, and F. W. Albert. Simultaneous quantification of mRNA and protein in single cells reveals post-transcriptional effects of genetic variation. *Elife*, 9, Nov 2020.

[59] A. N. Nguyen Ba, K. R. Lawrence, A. Rego-Costa, S. Gopalakrishnan, D. Temko, F. Michor, and M. M. Desai. Barcoded bulk QTL mapping reveals highly polygenic and epistatic architecture of complex traits in yeast. *Elife*, 11, Feb 2022.
